# Multipurpose Polymer Bragg Grating-Based Optomechanical Sensor Pad

**DOI:** 10.3390/s19194101

**Published:** 2019-09-23

**Authors:** Steffen Hessler, Patrick Bott, Stefan Kefer, Bernhard Schmauss, Ralf Hellmann

**Affiliations:** 1 Applied Laser and Photonics Group, University of Applied Sciences Aschaffenburg, Wuerzburger Straße 45, 63743 Aschaffenburg, Germany; stefan.kefer@th-ab.de (S.K.); ralf.hellmann@th-ab.de (R.H.); 2 Weber GmbH Pruefzentrum, Wailandtstraße 6, 63741 Aschaffenburg, Germany; p.bott@webergmbh.de; 3 Institute of Microwaves and Photonics, University of Erlangen-Nuremberg, Cauerstraße 9, 91054 Erlangen, Germany; bernhard.schmauss@fau.de

**Keywords:** Bragg grating, EpoCore, TPX, guitar pickup, accelerometer, vital sign sensor

## Abstract

Flexible epoxy waveguide Bragg gratings are fabricated on a low-modulus TPX™ polymethylpentene polyolefin substrate for an easy to manufacture and low-cost optomechanical sensor pad providing exceedingly multipurpose application potentials. Rectangular EpoCore negative resist strip waveguides are formed employing standard UV mask lithography. Highly persistent Bragg gratings are inscribed directly into the channel waveguides by permanently modifying the local refractive indices through a well-defined KrF excimer laser irradiated +1/-1 order phase mask. The reproducible and vastly versatile sensing capabilities of this easy-to-apply optomechanical sensor pad are demonstrated in the form of an optical pickup for acoustic instruments, a broadband optical accelerometer, and a biomedical vital sign sensor monitoring both respiration and pulse at the same time.

## 1. Introduction

Optical sensor principles continuously gain increasing interest in mechanical sensing applications since they feature inherent electromagnetic immunity in harsh environments as well as precise, multipurpose, and multiplexing measurement capabilities [1,2,3,4]. Especially, optical sensor technology based on Bragg gratings (BGs) is an ever-growing, popular, and robust tool of choice for various mechanical sensing tasks such as long-term structural health monitoring in general [5,6,7], strain sensing of composite materials in particular [8,9], and even biomedical vital sign monitoring [10,11,12].

Basically, an elementary Bragg grating comprises a periodic refractive index (RI) modulation inside an optical waveguide core. The narrow-band reflection signal resulting from constructive interference of the reflected waveguide mode at the periodic Bragg grating planes is defined as the Bragg wavelength $\lambda_{B}$ according to

(1) $${m\;\lambda }_{B}{=2\;n}_{\mathrm{eff}}\;\Lambda .$$

Here, $n_{\mathrm{eff}}$ represents the effective refractive index of the supported optical waveguide mode, Λ refers to the grating period and m is an integer describing the order of the optical grating’s reflection [13,14].

The exact Bragg wavelength is intriguingly sensitive to environmental conditions either altering $n_{\mathrm{eff}}$ or Λ thus providing the Bragg grating’s sensor signal. Characteristically, Bragg gratings feature a single fiber connection together for both input and output signal light paths only, being superior to other optical sensor types (e.g., Mach–Zehnder interferometer, Fabry–Pérot cavity) inevitably requiring an additional, elaborate transmission signal connection.

In contrast to the commonly employed Fiber Bragg gratings (FBGs), planar waveguide Bragg gratings offer a vast freedom of sensor design, which is synergized in particular by the use of polymer materials. Regarding their widely tunable material properties, optical polymers enable efficient optomechanical sensing of both compressive and tensile strain while maintaining low material costs and an easy fabrication process. Numerous studies on polymer planar Bragg gratings for sensing purposes have been published covering the prevalently employed grating materials, such as polymethylmethacrylate (PMMA) [15,16,17], cyclo-olefin copolymers (COC) [18,19,20], Ormocer^®^ hybrid polymers [21,22,23] as well as the epoxy-based photoresist EpoCore [24,25,26,27]. However, all of the proposed sensors’ performances are limited to specially designed optomechanical measurement applications only.

In this contribution, we therefore present an easy-to-use flexible Bragg grating sensor pad comprising a novel and advantageous polymer combination allowing reliable and highly versatile optomechanical sensing. Highly stable EpoCore (RI @1550 nm n_EpoCore_ ≈ 1.573) waveguide Bragg gratings are fabricated on a low modulus TPX™ (RI @1550 nm n_TPX_ ≈ 1.454) polymethylpentene substrate providing strong light confinement, i.e., strong optical waveguiding properties owing to the pronounced refractive index contrast. The sensor pad’s compelling adaptable usage is demonstrated by means of an acoustic instrument pickup, an optical accelerometer device as well as an opto-medical vital sign sensor capable of measuring respiration, pulse, and body temperature health signs in electromagnetic harsh environments (e.g., in magnetic resonance imaging) simultaneously.

## 2. Materials and Methods

**Figure 1:**
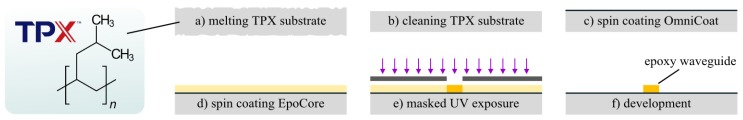
Schematic fabrication process of the flexible epoxy-based Bragg grating mechanical sensor device using high-elastic TPX™ substrate material.

Figure 1 schematically describes the fabrication process of the polymer sensor pad. Commercially available TPX™ polymethylpentene sheets (“Transparent Polymer X”, polymer grade DX845) of 150 mm × 150 mm dimension and a thickness of 0.5 mm are purchased (Goodfellow GmbH, ME311250) and cut to sample sizes of 15 mm × 25 mm. The raw TPX™ sheets are supplied with an undesirably high surface roughness, i.e., low optical quality necessitating a previous flattening step by exploiting the polymer’s dominant thermoplastic properties. Hence, the polymer samples are clamped between standard microscope slides and tempered at 245 °C on a hotplate (glass transition temperature of DX845 is T_G_ = 232 °C, [28]) efficiently eliminating surface roughness (Figure 1a). After cleaning the samples by purging in subsequent acetone, 2-propanol, and nitrogen media (Figure 1b), another essential surface pretreatment is accomplished by spin coating a thin adhesion promotion layer (30 s at 1000 rpm, soft bake for 120 s at 120 °C, Figure 1c) of OmniCoat™ (MicroChem). EpoCore 5 epoxy photoresist (Micro Resist Technology) is applied to the prepared TPX™ substrates, spin coated at 3000 rpm and soft baked at 90 °C for 300 s (Figure 1d). The epoxy layer is microstructured by the use of conventional i-line mask lithography (Figure 1e) with a UV dose of 200 mJ/cm^2^ (EVG620 mask aligner equipped with Osram HBO 350 W/S UV lamp) precisely defining straight epoxy waveguides on TPX™ after development in propylene glycol methyl ether acetate (PGMEA) for 60 s under permanent agitation (Figure 1f) [29].

Robust Bragg gratings with a maximum length of 5 mm are integrated into the center part of the epoxy waveguides on the pad by KrF excimer laser irradiation through a uniform +1/-1 phase mask increasing the waveguide’s local refractive index. The optimized laser writing conditions of Bragg gratings in EpoCore waveguides have already been described elsewhere in detail [12,15]. Finally, a single mode glass fiber pigtail is accurately glued (Norland NOA76) to the polished epoxy waveguide facet of each sensor sheet in order to connect to the 1000 Hz dynamic Bragg grating interrogation system (si155 HYPERION, Micron Optics).

## 3. Results and Discussion

### 3.1. Bragg Grating Reflection Spectrum

**Figure 2:**
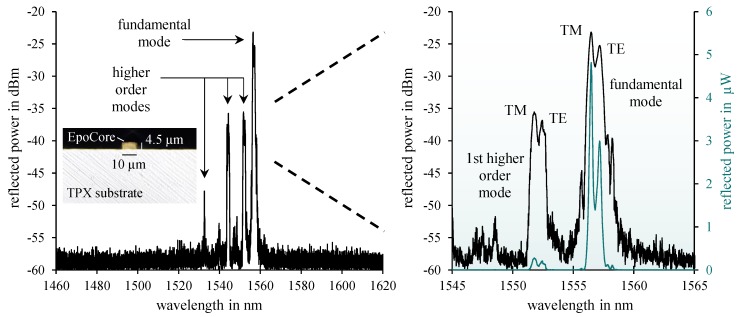
Typical reflection spectrum of an epoxy-based optomechanical Bragg grating sensor device. Multimode reflection peaks as well as distinctive peak splitting in TE and TM modal reflections can clearly be observed. The micrograph shows a cross sectional view of the waveguide Bragg grating.

**Figure 3:**
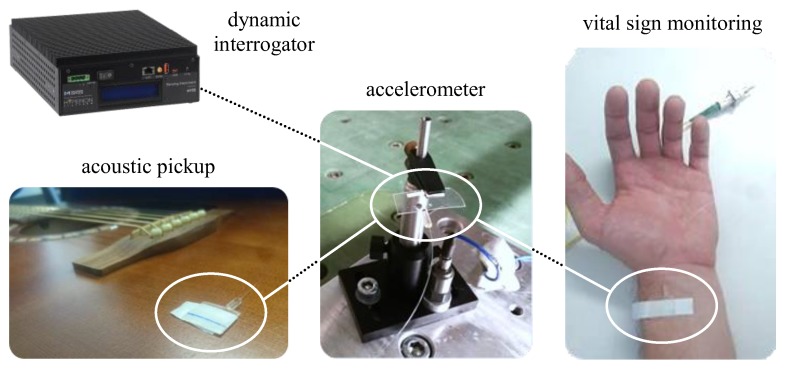
Tested optomechanical application scenarios of the flexible Bragg grating sensor device by the use of a Micron Optics HYPERION si155 dynamic interrogation system providing sensor signal rates up to 1000 Hz.

In Figure 2, a typical reflection spectrum of the fabricated epoxy Bragg grating with a grating period of Λ = 990 nm is depicted employing dynamic Bragg wavelength interrogation. A low signal baseline of about −58 dB is obtained consistently over the considerably broad interrogation range from 1460 nm to 1620 nm. A pronounced fundamental mode as well as higher order mode reflections can clearly be observed around 1550 nm due to the multimode nature of the ~10 µm wide and ~4.5 µm high rectangular strip waveguide. Both fundamental and higher order mode reflections exhibit a distinct peak splitting into TE (transverse electric) and TM (transverse magnetic) modal reflection signals indicating severe birefringence effects inside the polymer material combination. Considering the huge mismatch of thermal expansion coefficients (CTE) between TPX™ (CTE_TPX_ ≈ 117 ppm/K) and EpoCore (comparable CTE value of similar epoxy-based photoresist SU8 CTE_SU8_ ≈ 52 ppm), this remarkable peak splitting is—beside inherent material birefringence—most likely due to mechanical stresses introduced during fabrication which expresses in the form of stress induced birefringence. The differing effective refractive index values for the fundamental TE and TM mode reflection amount to n_eff_ (TE) = 1.57295 and n_eff_ (TM) = 1.57221. All observed signals turn out to be detectable in a highly stable and reproducible way even in mechanically strained condition. Consequently, all existing Bragg grating peaks and their mechanically induced wavelength shifts in time (by changing $n_{\mathrm{eff}}$ and Λ) may be utilized as optomechanical sensor signals in combination with a dynamic Bragg interrogation system for versatile mechanical measurement purposes such as acoustic vibration, acceleration as well as vital sign monitoring as illustrated in Figure 3.

### 3.2. Sensor Application as an Optical Pickup for Acoustic Instruments

**Figure 4:**
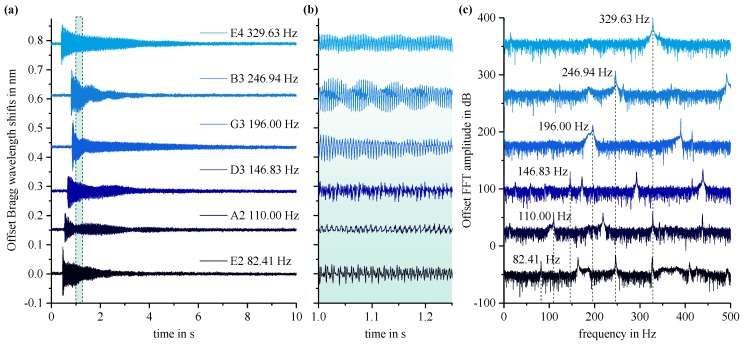
Response of a Bragg grating sensor applied to the flat top of an acoustic guitar. (**a**) Bragg wavelength shifts for plucked guitar strings in standard tuning; (**b**) enlarged view of the sensor’s recorded audio signals; (**c**) Fast Fourier transformation (FFT) spectra of all six signals verifying the fundamental and additional harmonic overtone frequencies.

The sensor pad’s TPX™ substrate backside is equipped with a conventional double-sided scotch tape to establish a firm connection to the flat top of a steel-string acoustic guitar body (Art & Lutherie Cedar Dreadnought). In this way, an efficient mechanical transfer of the acoustic vibrations to the Bragg grating is ascertained. The guitar strings are precisely tuned to standard tuning (E2-A2-D3-G3-B3-E4). All six strings are plucked individually, which characteristically stimulates acousto-mechanical oscillations of the guitar’s flat top and the resultant Bragg wavelength shifts of the sensor’s fundamental mode Bragg reflection are recorded. In Figure 4a, the temporal sensor signals for each string are shown together with an enlarged view in Figure 4b illustrating the recorded audio signals. The applied sensor’s signals show typical sinusoidal response with distinct maximum deflections around ~100 pm confirming exact and delicate recording capabilities. Fast Fourier transformation (FFT) of every single audio signal reveals the precise frequencies for the fundamental tone as well for the associated harmonic overtones (in terms of integral multiple of the fundamental frequency).

### 3.3. Sensor Application as an Optical Accelerometer

In order to acquire the precise sensitivity to acceleration, the sensor pad is clamped unilaterally on top of a mechanical testing machine providing exactly calibrated acceleration values. In response to a sinusoidal acceleration stimulus, the freehanging sensor end is deflected equivalently, thus generating measurable Bragg wavelength shifts by altering $n_{\mathrm{eff}}$ and Λ. Thereby, the reproducible peak splitting effect due to the present birefringence is fully utilized to obtain corresponding sensitivity curves for both the fundamental TE and TM mode reflection.

**Figure 5:**
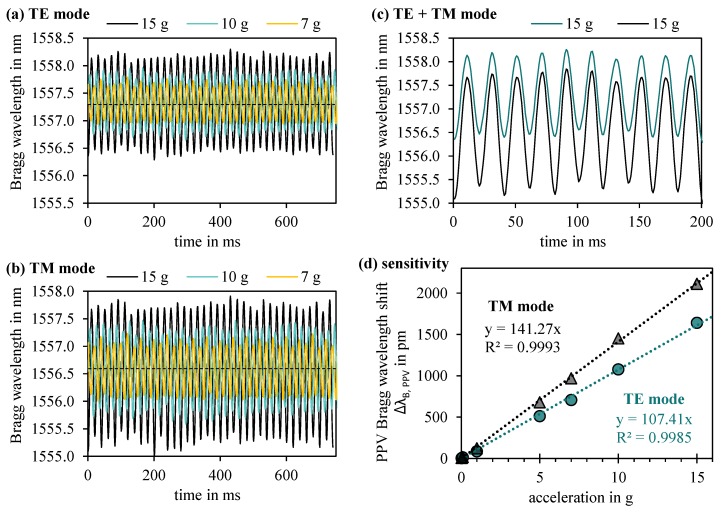
Sensor response to different impinged acceleration values with a precisely calibrated frequency of 50 Hz. (**a**) Temporal Bragg wavelength signal of the TE mode; (**b**) temporal Bragg wavelength signal of the TM mode; (**c**) well-separated TE and TM modal reflection signals even at high acceleration value of 15 g; (**d**) linear accelerometer sensitivities of the fundamental TE and TM modal reflection.

Figure 5 summarizes the pronounced and consequent sinusoidal shifts of the Bragg wavelengths for fundamental TE (Figure 5a) and TM (Figure 5b) modal reflection exemplarily on 15 g, 10 g and 7 g acceleration stimulation with a frequency of 50 Hz attesting the sensor pad a fast and meticulous response. Direct comparison of the two modal sinus signals evidently displays a significantly higher sensitivity of the TM mode Bragg reflection to acceleration. Figure 5c corroborates immaculate, non-interfering signal resolution between TE and TM modal reflection even at a comparably high acceleration impact of 15 g. In Figure 5d, the sensor pad’s modal-dependent sensitivity is expressed in terms of averaged peak-to-peak values (PPV) of the sinusoidal response curves. Both TE and TM mode indicate linear reaction to acceleration with a sensitivity of 107 pm/g and 141 pm/g, respectively. We attribute the intriguingly higher acceleration sensitivity of the TM mode signal to the severe birefringent mechanisms of the waveguide design.

### 3.4. Sensor Application as an Optical Vital Sign Monitor

In order to employ the sensor pad for vital sign monitoring purposes in an easy-to-apply way, the TPX™ substrate backside, in turn, is equipped with a double-sided surgical tape to facilitate full skin contact to the test person’s body part of interest. In principal, for medical pulse sign recording several sensor positions near specific major arteries such as aorta, radial artery, carotid, and femoral artery may be considered to cause a change of the effective refractive index and the grating period. 

**Figure 6:**
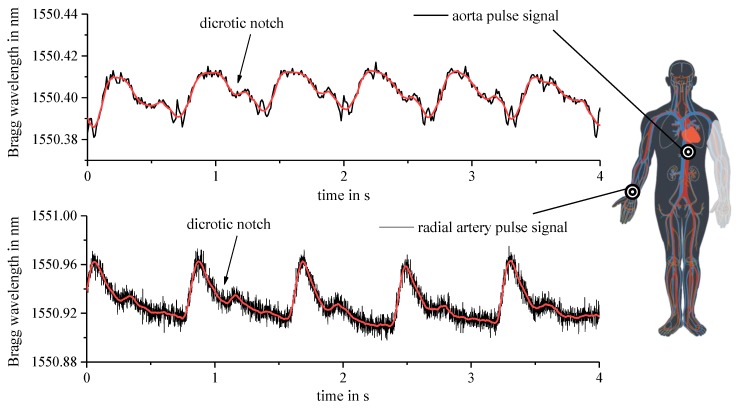
Epoxy-based sensor pad applied for monitoring aorta and radial artery pulse signs of a test person (draft of circulatory system based on [30]).

The developed optomechanical sensor’s performance in pulse measurement of a test person is depicted in Figure 6. At first, the sensor pad is placed at the center of the torso for aorta pulse measurement. After that, the radial artery pulse signal is recorded likewise for comparison. The fundamental TE mode reflection is tracked by the dynamic interrogation system. Both observed pulse wave forms agree with the expected shape from a medical point of view. Most notably, all pulse signals in both diagrams are featured with the characteristic dicrotic notch in the pulse wave from heart valve activity indicating accurate sensor operation. The frequencies of the exemplified pulse measurements amount to 90 bpm (aorta) and 75 bpm (radial artery), which may be temporally monitored by continuous averaging.

**Figure 7:**
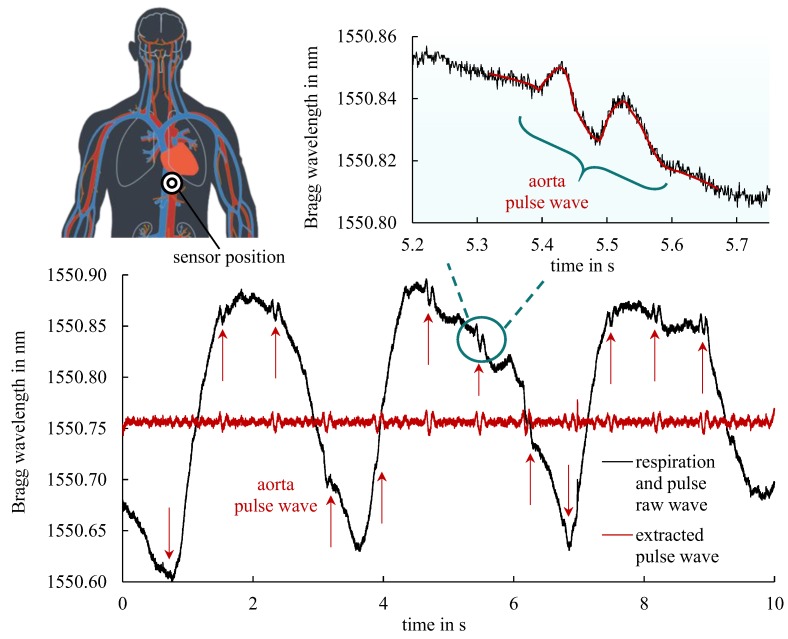
Sensor pad applied to the aorta region for simultaneous vital sign monitoring of respiration and pulse waves (draft of circulatory system based on [30]). The inset clearly indicates a superimposed pulse signal wave which is readily extracted by high pass filtering (red curve).

Aiming at simultaneous vital sign monitoring of both respiration and pulse, the sensor pad is placed below the left chest side in the xiphoid level area enabling additional mechanical sensing of the abdominal wall movement. The sensor’s special position is schematically depicted in Figure 7. Accordingly, the resulting recorded Bragg wavelength signal comprises the respiration wave as well as the superimposed pulse wave from aorta blood pressure. The huge change of the Bragg wavelength in the order of hundreds of picometers represents the test person’s respiration cycle. The typical form of the periodic pulse wave can be clearly recognized in the enlarged inset. Consequently, by the use of suitable filter algorithms the pulse frequency as well as the respiration frequency can be determined independently and monitored concurrently in a single measurement.

## 4. Conclusions

In this work, an innovative, multipurpose optomechanical sensor pad comprising robust epoxy-based EpoCore waveguide Bragg gratings on a specially prepared low-modulus TPX™ polymethylpentene substrate is introduced. The presented simple lithographic batch fabrication process along with the low material costs of the full polymer sensor sheet offer a vast number of potential applications. A single fiber connection to the optical waveguide Bragg grating element suffices the dynamic interrogation of the reflected Bragg wavelength shifts due to mechanical stimuli, which makes the sensor pad easy-to-apply. The sensor pad’s general optomechanical performance and versatile application suitability in terms of an acoustic pickup, an accelerometer, and a vital sign monitor especially for harsh electromagnetic environments is characterized in detail and confirmed thoroughly imperatively demanding further development.
